# Panaxynol, a bioactive component of American ginseng, targets macrophages and suppresses colitis in mice

**DOI:** 10.18632/oncotarget.27592

**Published:** 2020-06-02

**Authors:** Anusha Chaparala, Deepak Poudyal, Hossam Tashkandi, Erin E. Witalison, Alexander A. Chumanevich, Jenna L. Hofseth, Ivy Nguyen, Olivia Hardy, Douglas L. Pittman, Michael D. Wyatt, Anthony Windust, Elizabeth A. Murphy, Mitzi Nagarkatti, Prakash Nagarkatti, Lorne J. Hofseth

**Affiliations:** ^1^Department of Drug Discovery and Biomedical Sciences, College of Pharmacy, University of South Carolina, Columbia, SC, USA; ^2^Laboratory of Human Retrovirology and Immunoinformatics, Leidos Biomedical Research Inc., Frederick National Laboratory for Cancer Research, Frederick, MD, USA; ^3^Department of Biological and Biomedical Sciences, Julius L. Chambers Biomedical/Biotechnology Research Institute, North Carolina Central University, Kannapolis, NC, USA; ^4^Measurement Science and Standards, National Research Council, Ottawa, ON, Canada; ^5^Department of Pathology, Microbiology, and Immunology, School of Medicine, University of South Carolina, Columbia, SC, USA

**Keywords:** inflammatory bowel diseases, ulcerative colitis, American ginseng, panaxynol, macrophages

## Abstract

Ulcerative colitis has a significant impact on the quality of life for the patients, and can substantially increase the risk of colon cancer in patients suffering long-term. Conventional treatments provide only modest relief paired with a high risk of side effects, while complementary and alternative medicines can offer safe and effective options. Over the past decade, we have shown that both American ginseng and its hexane fraction (HAG) have anti-oxidant and anti-inflammatory properties that can suppress mouse colitis and prevent colitis-associated colon cancer. With the goal of isolating a single active compound, we further fractionated HAG, and found the most abundant molecule in this fraction was the polyacetylene, panaxynol (PA). After isolating and characterizing PA, we tested the efficacy of PA in the treatment and prevention of colitis in mice and studied the mechanism of action. We demonstrate here that PA effectively treats colitis in a Dextran Sulfate Sodium mouse model by targeting macrophages for DNA damage and apoptosis. This study provides additional mechanistic evidence that American ginseng can be used for conventional treatment of colitis and other diseases associated with macrophage dysfunction.

## INTRODUCTION

Inflammatory bowel diseases (IBDs), including ulcerative colitis (UC) and Crohn’s disease (CD), are debilitating illnesses that significantly affect patients’ lifestyle and carry a high colon cancer risk. IBD prevalence is particularly high in North America and Europe (affecting 3.8 million people), with an economic burden of $30–$45 billion [1–4]. Of note, incidence has been increasing for both males and females over the past 20 years [5]. Frustratingly, conventional treatments of IBD patients have modest outcomes with 20% of patients not responding to anti-TNFα antagonists [6], and toxicity leads to dangerous side effects. As such, about half of all IBD patients (millions) turn to complementary and alternative medicines (CAMs). Although CAMs have been used for thousands of years, there is a gap in our knowledge of the mechanisms that support their effectiveness. Understanding these mechanisms will not only lead to standardized and more efficient treatment for IBD outside of toxic FDA-approved drugs but will also better our understanding of the potential applications of CAMs for other diseases with similar mechanisms.

Inflammation generally occurs as an acute response to an injury and infection. This response is initiated by the activation of sentinel immune cells, such as macrophages and dendritic cells that reside around the injured or infected area, which then release chemokines and cytokines and may further recruit other immune cells [7–9]. Control of the immune response to infection is essential to preventing it from becoming a chronic condition. This control is done through apoptosis of immune cells via the tumor suppressor protein 53 (p53) [10, 11]. Low p53 levels in macrophages were observed to be a cause for higher expression of NF-κB-targeted, pro-inflammatory cytokines such as interleukin-6 (IL-6) and tumor necrosis factor (TNF), which are involved in chronic inflammation such as IBD [12–14].

The natural herb, American ginseng (*Panax quinquefolius*; AG), improves mental performance and detrimental end points associated with diseases, such as cardiovascular disease, diabetes, and influenza [15, 16]. Over the past decade, we have shown that AG has anti-oxidant and anti-inflammatory properties and is able to suppress mouse colitis and prevent colon cancer associated with colitis [11, 17, 18]. Using bioassay-guided fractionation, we have shown that a hexane fraction of AG was particularly potent in this capacity [19–21].

Polyacetylenes are a distinct group of naturally occurring products, whose numerous pharmacological properties have been recognized [22, 23]. PA ([3(R)-(9Z)-heptadeca-1, 9-dien-4, 6-diyn-3-ol]; falcarinol) is a bioactive member of this family. It has been identified in both traditional herbal medicines, such as AG, and dietary plants, e. g., carrots, celery, and fennel [24]. Interestingly, PA has been shown to have anti-cancer properties [24–27] and neuroprotective effects [28–30]. However, there remains an unanswered question regarding PA’s potential as an anti-inflammatory molecule and, therefore, its capacity to suppress chronic inflammatory diseases, such as UC. Here, we hypothesize that the most abundant single molecule ingredient of HAG is the active component of reducing inflammation in a mouse model and that this molecule targets macrophages (mФ) for apoptosis resulting in the suppression of colitis in mice.

## RESULTS

### Panaxynol is the most abundant and a potent anti-inflammatory molecule in AG

**Figure 1:**
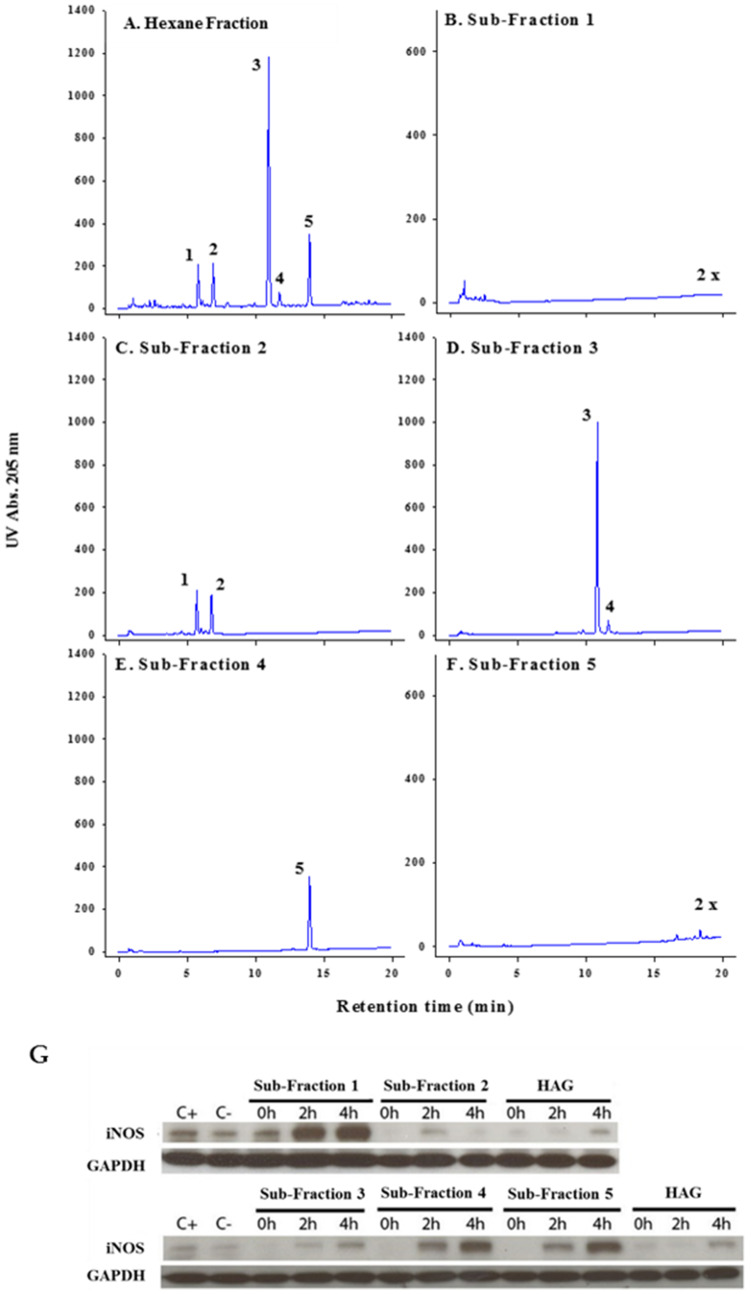
Isolation and characterization of various sub-fractions of HAG. (**A**–**F**) LC-UV/DAD analysis of Hexane fraction and each sub-fraction. F1 to F5 represent the collected fractions, 4 minutes each. Peak identities: 1. Panaxydiol, 2. Panaxydol, 3. Panaxynol, 4. linolenic acid, 5. linoleic acid Column C-18 2.1 × 100 mm, 1 μl injection of a 5 mg mL^-1^ (whole) or equivalent fraction, gradient 55% to 90% acetonitrile/water in 15 minutes; hold 5 minutes; re-equilibration 10 minutes. Note: The scale magnification for sub-fractions 1 and 5 is 2×. (**G**) Effect of HAG and different sub-fractions of HAG on IFNγ-induced iNOS expression. ANA-1 mouse mФ were incubated for 12 hours with HAG or the indicated sub-fractions (10 μg/ml), washed, then exposed to IFNγ (10 ng/ml) for 0, 2, and 4 hours. C+ indicates the positive control, which is ANA-1 cells stimulated by IFNγ, and then incubated with media.

We have previously shown that AG and HAG are effective in the treatment of colitis and prevention of colon cancer [11, 17–21]. We have also demonstrated that fatty acids and polyacetylenes are both components in AG and HAG [19]. In moving forward, to better understand the active components of HAG, we sub-fractionated this fraction of AG using liquid chromatography with UV/diode array detection (LC-UV/DAD) (Figure 1A). Fraction 1 (< 10% of the whole HAG) contains multiple minor components including two minor polyacetylenes tentatively identified based on UV spectra (Figure 1B). Fraction 2 (30% of HAG) contains two major polyacetylenes, panaxydiol (peak1) and panaxydol (peak 2), and four minor polyacetylenes tentatively identified based on UV spectra (Figure 1C). Fraction 3 (24% of HAG) contains a major polyacetylene, PA (peak 3), and a fatty acid, linolenic acid (peak 4) (Figure 1D). Fraction 4 (27% of HAG) contains linoleic acid (peak 5) and no detectable polyacetylenes (Figure 1E). F5 (10%) contains minor fatty acids including saturates, and no polyacetylenes (Figure 1F).

The subfractions were used to treat ANA-1 mФ cell-line after they were polarized to M1 using interferon γ (IFNγ, 10 ng/ml) for 0, 2, and 4 hours. Using western blot, we show that fractions 2 and 3, the only fractions containing major polyacetylenes, suppress inducible nitric oxide synthase (iNOS) expression (Figure 1G), which is predictive of colitis suppression [17, 19]. Of the three major polyacetylenes in fractions 2 and 3 of HAG, PA was the most abundant (10.2%) molecule.

### Panaxynol is effective as a treatment for colitis in Dextran Sulfate Sodium (DSS) mouse model

Following the isolation of PA from HAG, and an initial screening (iNOS suppression *in vitro* [31]), we tested the efficacy of this compound in the prevention and treatment of DSS-induced mouse colitis. The PA doses were equated to reflect the percentage composition of PA in HAG. In the prevention model, where mice were treated with PA for a week before the induction of colitis using DSS (Supplementary Figure 1A), treatment with PA did not inhibit colitis in mice when compared to the control group. Moreover, there was a marginal increase in the inflammation score with the highest dose of PA (Supplementary Figure 2A–2B) when compared to the vehicle group. This means that treatment with PA prior to DSS treatment slightly exacerbated DSS-induced colitis, indicating the inability of PA to act as a preventative method.

**Figure 2:**
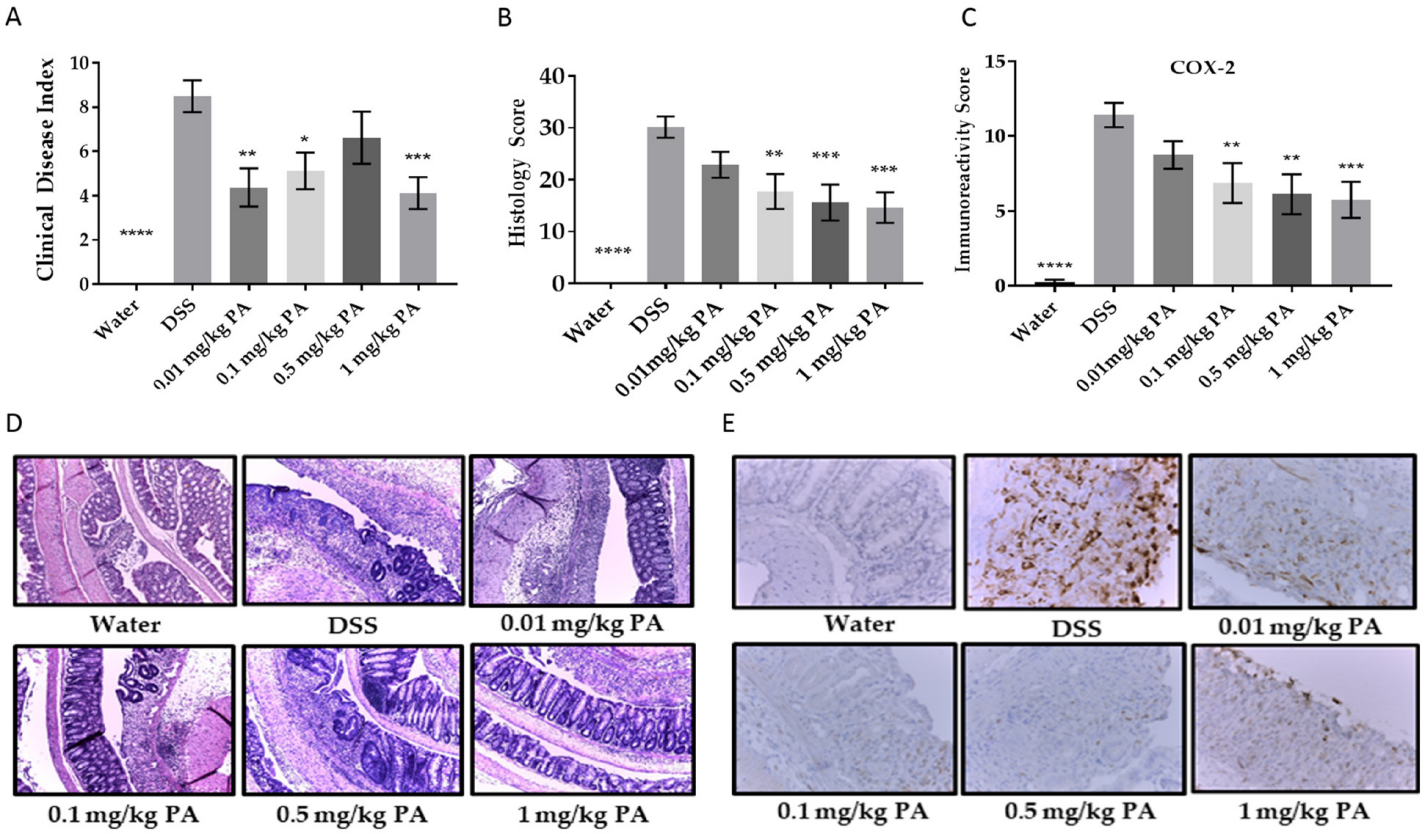
Panaxynol suppresses DSS-induced colitis in mice. (**A**) Representative images (magnification–100×) of histological sections from 3 groups; water, DSS only and highest dose of PA (1 mg/kg/day). (**B**) Inflammation scores obtained from H & E slides of the colon cross-sections. (**C**) Representative images of sections stained for COX-2 (magnification – 400×). (**D**) Immunoreactivity score (IRS) of COX-2 from IHC staining. (**E**) Clinical Disease Index (CDI) accounts for weight loss, blood in stool and stool consistency (*n* = 8). Values represent mean ± SEM. One-way ANOVA followed by Dunnett’s test was used for comparison between samples. *p*-value when compared to DSS group is indicated by: ^*^ = < 0.05, ^**^ = < 0.01, ^***^ = < 0.001, ^****^ = < 0.0001.

Excitingly, PA was very effective in the treatment model of colitis (Supplementary Figure 1B), where colitis was induced with DSS for a week followed by PA treatment. PA significantly decreased the Clinical Disease Index (CDI) (Figure 2A) and the inflammation score (Figure 2B, 2D) in a dose-dependent manner. Colonic inflammation from PA-treated mice was limited to the distal end of the colon, while in the vehicle group, inflammation involved a larger area. To examine a biomarker of inflammation, we tested each colon section for cyclooxygenase-2 (COX-2) immunoreactivity using immunohistochemistry. There was a decreased expression of COX-2 with PA treatment (Figure 2C, 2E). Taken together, the results are consistent with the hypothesis that PA can be used to treat mouse colitis. To note, we monitored the weights of the mice over the course of the experiment and did not observe any unexpected weight loss even with the highest dose of PA, indicating the non-toxic nature of PA.

### Panaxynol targets macrophages for DNA damage *in vitro*

**Figure 3:**
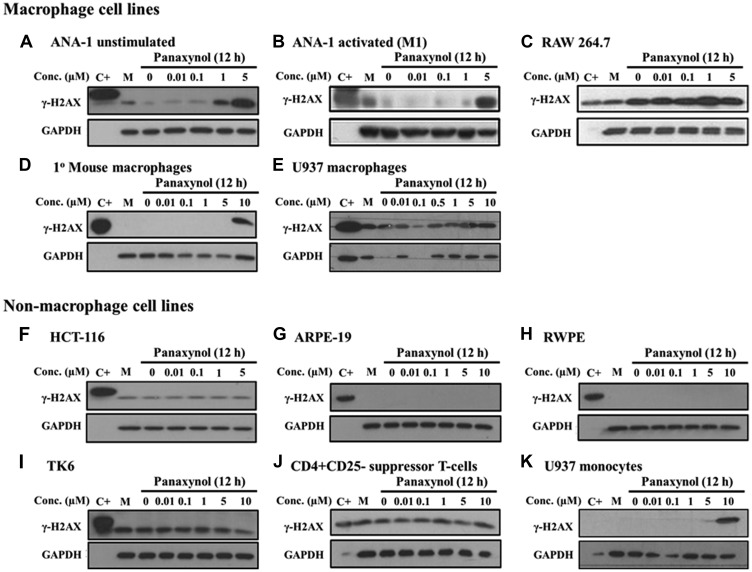
Panaxynol induces γ-H2AX in macrophages, but not in other cell types. All cell types were treated with PA at specified doses for 12 hours. Activated mФ were generated by treating with IFNγ (10 ng/ml for 8 hours) prior to PA treatment. U-937 cells were treated with 10 ng/ml PMA for 24 hours for differentiation into mФ. (**A**–**E**) MФ showed increased DNA damage with doses starting from 1 μM, as shown by the increase in the expression of γ-H2AX, a sensitive marker of DNA damage. (**F**–**J**) Non-macrophage cell lines, including other immune cells (i. e. lymphoblasts and T cells) and epithelial cell lines, did not show any change in the protein expression of γ-H2AX and (**K**) U-937 cells which are monocytes were more sensitive than U-937 mФ. M - cell culture media.

In an effort to identify the mechanism of action of PA, we studied the structure and observed that PA is a hydrophobic compound with several sites of potential modification that could convert it to a DNA alkylating agent (Supplementary Figure 3). The hydroxide at the 3-position (C3) can be converted to an α, β unsaturated aldehyde, which is a Michael acceptor, while the double bond between the 9 and 10 position could potentially be converted to an epoxide. Furthermore, the hydroxide group on C3 can react with the amino group of nucleic acids and alkylate DNA. We, therefore, screened multiple cell types for PA-induced DNA damage. Strikingly, PA caused DNA damage, as identified by phosphor-H2A histone family, member X (γ-H2AX) expression. However, γ-H2AX induction only occurred in mФ cell lines. These included mouse mФ (ANA-1, Figure 3A–3B; RAW264.7, Figure 3C), primary peritoneal mouse mФ (Figure 3D), and human mФ (U-937 after differentiation using 10 ng/mL of phorbol-12-Myristate-13-Acetate [PMA], Figure 3E). For all non-macrophage cells (Figure 3F–3K), γ-H2AX induction was not seen up to 10 μM PA treatment. As well, when U-937 human monocytes were not differentiated to mФ, γ-H2AX induction was also not seen until 10 μM PA treatment (Figure 3K) when compared to induction at 1 μM in U-937 cells differentiated into mФ (Figure 3E). This indicated the specificity of DNA damage to mature mФ and not monocytes.

### Panaxynol selectively targets macrophages for apoptosis *in vitro* and *in vivo*

**Figure 4:**
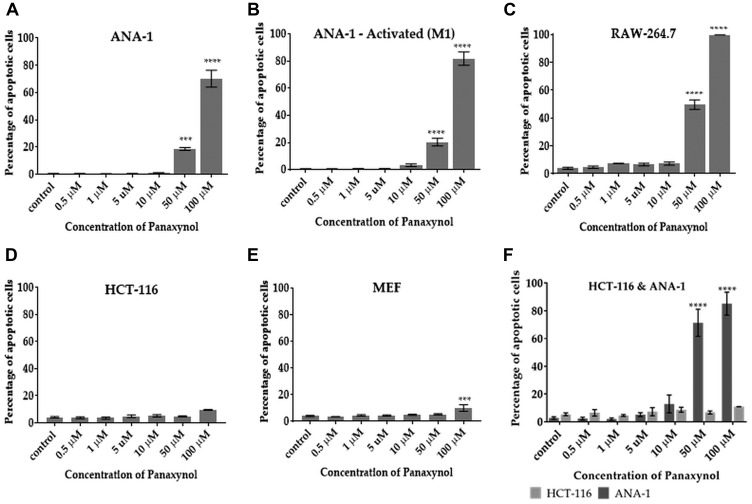
Panaxynol induces apoptosis in macrophages, but not in HCT-116 and MEF cells. Cells were treated with PA for 12 hours with indicated doses. PA significantly increased the percentage of apoptotic cells in (**A**) unstimulated ANA-1 cells at 50 μM (18%) and 100 μM (70%), (**B**) IFNγ stimulated ANA-1 cells at 10 μM (3.3%) and (**C**) RAW264.7 cells at 50 μM (50%) and 100 μM (99%). (**D**) PA had no significant apoptotic effect on HCT-116. (**E**) PA induced apoptosis in MEFs only at a high dose of 100 μM (9.5%). (**F**) In a co-culture experiment, PA caused apoptosis only in ANA-1 cells, but not HCT-116 cells. *p*-value indicated by; ^*^ = < 0.05, ^**^ = < 0.01, ^***^ = < 0.001, ^****^ = < 0.0001.

Based on the understanding that DNA damage is associated with apoptosis, we hypothesized that PA can selectively cause apoptosis in mФ. Results are consistent with this hypothesis in two macrophage cell lines (Figure 4A–4C). Apoptosis was minimal in other non-macrophage cells, including HCT-116 cells (Figure 4D) and mouse embryonic fibroblasts (MEFs) (Figure 4E). To examine whether PA selectively causes apoptosis in mФ in the presence of other cell types, we carried out a co-culture experiment with M1 polarized ANA-1 mФ and colon cancer cells (HCT-116). Figure 4F shows that PA causes apoptosis in ANA-1 mФ at significantly higher levels than in HCT-116 cells.

**Figure 5:**
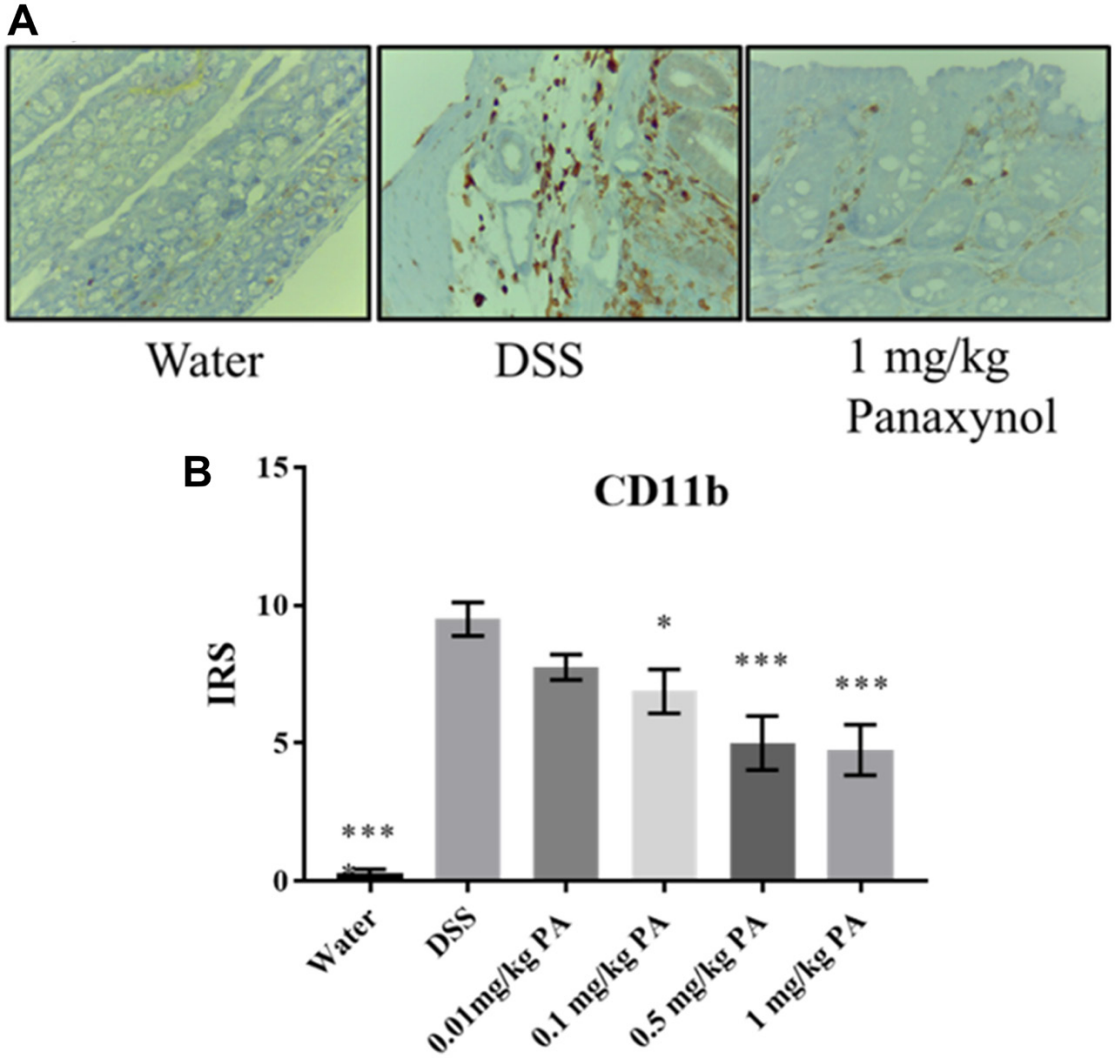
Panaxynol targets macrophages *in vivo*. Effect of PA on macrophages *in vivo*. (**A**) Representative images of sections stained for CD11b (magnification – 400×) (*N* = 8). (**B**) Immunoreactivity score (IRS) of CD11b from IHC staining. *p*-value indicated by; ^*^ = < 0.05, ^**^ = < 0.01, ^***^ = < 0.001, ^****^ = < 0.0001.

To confirm that PA targets mФ *in vivo*, we used colons from the DSS-induced colitis experiment to perform IHC for mФ. We used a CD11b antibody, which is a surface marker for mФ, and we observed that PA-treated colons have lower expression of CD11b when compared to the vehicle group, indicating that PA treatment decreased the number of mФ *in vivo* (Figure 5).

## DISCUSSION

Currently available treatments for IBD have multiple side effects and affect major organs like kidneys, liver (hepatitis), and pancreas (pancreatitis) [32]. Furthermore, immune targeting drugs, e. g., infliximab that targets the tumor necrosis factor (TNF) pathway, are broadly immunosuppressive with major side effects including a higher risk of non-Hodgkin’s lymphoma [33]. We have shown that AG treats colitis in mice [17]. However, it is composed of multiple ingredients with diverse effects, making it unfit for use as a mainstream drug. Upon examining the different extracts of AG, we identified HAG to be the most effective fraction in the treatment of colitis [19]. Further analysis examined the various components of HAG’s ability to suppress iNOS, an inflammatory response gene, in mФ. PA, apart from being the most abundant molecule in HAG, is also more effective than the whole HAG in suppressing iNOS expression in mФ that are polarized to M1 (pro-inflammatory). Hence, testing PA for the treatment of colitis is a natural step towards the identification of the bioactive component to treat colitis and prevent colon cancer.

The DSS mouse model is used due to its ability to produce inflammation in mice resembling UC and it is symptoms [34]. Therefore, consistent with our previous studies with AG and UC, we used DSS-induced mouse colitis model for studying the effect of PA on an inflammatory disease. We found that PA treats DSS-induced colitis in the mouse, as seen by decreased CDI, inflammation, COX-2 expression, and the halted weight loss in treated mice. There was no toxicity even at higher doses, as observed by the insignificant weight changes. In future experiments, we will examine the effect of PA on the liver and kidneys to further rule out toxicity.

One of the mechanisms by which AG and HAG treat colitis is by targeting immune cells for apoptosis [11, 20]. We also examined the structure of PA and identified it to be a hydrophobic compound, is a potential DNA-reactive alkylating agent. PA and its derivative, falcarindiol, have previously been shown to be protein-alkylating agents [35]. Furthermore, it has been shown that PA causes DNA damage in the colorectal cancer epithelial cell-line, CaCo2 [36]. It can be reasoned that the mechanism of action of PA can be via the induction of DNA damage. Our preliminary results show that PA causes DNA damage in multiple cell lines and that mФ are especially sensitive to DNA damage induced by PA. In fact, we have tested a range of doses (0.01 mg/kg–1 mg/kg, Figure 3) and demonstrated that PA is very effective at 0.1 mg/kg, which would translate to 6 mg for an average patient weighing 60 kg. This is an extremely low dose when compared to the immunosuppressive drugs currently available, placing PA a step above the other treatments for UC. Seeing that PA seems to target mФ specifically for apoptosis through DNA damage, we predict that this is the key component in PA’s anti-inflammatory effect.

PA, however, did not prevent colitis in mice. Furthermore, treatment with the highest dose of PA slightly increased the inflammation score when compared to the untreated mice. The resident mФ in the lamina propria of the intestine are anti-inflammatory and important for the maintenance of homeostasis. They clear any microbes and other stimuli that cross the epithelial cell barrier, mainly by phagocytosis, but do not secrete any cytokines [37]. Since PA targeted mФ before induction of colitis in the prevention model, the disease was more severe and PA was ineffective. This is consistent with previous studies that showed that depletion of mФ prior to induction of colitis resulted in exacerbated DSS-induced colitis [38]. However, upon initiation of UC, there is increased accumulation of pro-inflammatory mФ that secrete cytokines to enhance the inflammatory response. An overactive response by the mФ to the enteric microbiota at this stage greatly contributes to the pathogenesis of colitis [39]. Treatment with PA to target mФ at this stage was highly effective in suppressing colitis and emphasizes the effectiveness of PA in treating an autoimmune inflammatory disease. For that reason, PA may also work for CD or even rheumatoid arthritis [40].

The reason for the mФ being specifically targeted by PA is not completely understood. However, this property of PA would distinguish it from broadly immunosuppressive drugs that are currently on the market for the treatment of UC. Furthermore, PA (as compared to the hundreds of other potential CAMs currently used with success in animals) not only comes from a natural source, but as a single ingredient, allowing the potential to be standardized on its own, or in a cocktail. Future directions involve investigating whether PA can prevent colon cancer as the next natural step, since macrophage depletion not only decreases inflammation but also suppresses tumorigenesis in AOM-DSS-induced model of colitis induced colon cancer in mice [41]. Another direction to explore in the future is to determine if PA can achieve its anti-inflammatory effects in a CD mouse model.

## MATERIALS AND METHODS

### Identification and isolation of panaxynol

Characterization of HAG and extraction of PA were carried out by our collaborator, Dr. Anthony Windust at the National Research Council (Ottawa, ON, Canada). The method for characterization and analysis of HAG has been described in detail previously [19]. Briefly, for characterization of bioactive components of HAG, this fraction was sub-fractionated through preparative, reverse-phase HPLC, where the HAG was divided into 5 sub-fractions based on elution time (4 minutes each). The fractions were collected over 6 repeat runs (6 × 50 mg injected) and evaporated to dryness. A comparative analysis by analytical scale LC-UV of both the whole and each sub-fraction was performed to confirm identities of constituents in each sub-fraction.

PA was isolated and purified from *Panax quinquefolius* grown on the Harper Ranch, Kamloops, BC, Canada. The method of extraction and purification of PA has been previously described [31]. Briefly, dried root of four-year-old AG was dissolved in ethanol and the organic layer was concentrated using vacuum centrifuge to yield dark brown oil. This extract was further separated using flash chromatography and the fractions containing PA were dried to yield crude PA. The crude PA was then subjected to multiple passes of chromatography and the purity of the final extract was validated using liquid chromatography with UV diode array detection (LC-UV-DAD). Purified PA was dissolved in 95% ethanol for use in *in vitro* and *in vivo* experiments.

### Cell lines and reagents

All cells were maintained in appropriate media for each cell-line recommended by ATCC supplemented with 10% New Born Calf serum (NBCS) (Biofluids, Rockville, MD), penicillin (10 U/ml) and streptomycin (10 μg/ml, Biofluids) at 37°C in a humidified chamber with 5% CO_2_ atmosphere. Experiments with PA were carried out by treating the cells with indicated concentrations of PA dissolved in appropriate media with 0.1% NBCS. For polarization to M1 type mФ, ANA-1 cells were exposed to 10 ng/ml interferon-γ (IFNγ) for 8 hours (R&D Systems, Minneapolis, MN). For differentiation of U-937 monocytes into mФ, cells were treated with 10 ng/ml phorbol 12-myristate 13-acetate (PMA) (Sigma; P1585) for 24 hours. After replacing with fresh media containing no PMA, the cells were allowed to grow for 48 hours before treatment with PA. CD4+CD25- cells were isolated from the spleens of C57BL/6 mice as previously described [20]. Briefly, the mФ and B cells were depleted before isolation of CD4+CD25- T cells using MACS separator along with CD4 and CD25 microbeads (Miltenyi Biotec, Auburn, CA).

### Western blot analysis and antibodies

Phospho-Histone H2AX (Ser139) (cat #9718S), phospho-p53 (Ser15) (cat #9284S), and GAPDH (D16H11) (cat #5174S) rabbit monoclonal primary antibodies (1:1000 dilution); and horseradish peroxidase conjugated anti-rabbit secondary antibody (7074S) (1:2000 dilution) were purchased from Cell Signaling Technology, Danvers, MA. Primary antibody incubations were carried out overnight at 4°C Secondary antibody incubations were carried out at room temperature for 1 hour. The Western blot signal was detected by Pierce ECL Western Blotting Substrate (Thermo Scientific, Rockford, IL) and developed onto Hyperfilm (GE Healthcare Life Sciences, Pittsburgh, PA) or imaged using Bio-Rad ChemiDoc Imager.

### Flow-cytometric TUNEL analysis

TUNEL (Terminal deoxynucleotidyl transferase dUTP nick end labeling) was performed using Fluorescein *in situ* cell death detection (cat #11684795910, Roche Diagnostics, IN). Briefly, cells were incubated in 0.1% NBCS supplemented media containing appropriate concentrations of PA or vehicle. Cells were harvested after 12 hours of treatment and TUNEL assay was performed as described by the vendor with DNAse from Sigma-Aldrich as positive control. TUNEL positive cells were detected and quantified using Beckman Coulter F500 Flow Cytometer and CXP software.

### *In vivo* experiments

DSS (MW 36000–50000) obtained from International Laboratories USA (San Francisco, CA) was used to induce colitis in mice. 8–10 weeks old C57BL/6 mice were obtained from Jackson Laboratories (Bar Harbor, ME) and maintained in a suitable environment according to the Institutional Animal Care and Use Committee (IACUC) standards. The care and usage of the mice were monitored by Animal Resource Facility (ARF) at the University of South Carolina, Columbia. This study was approved by IACUC (Animal Use Protocol # 2178).

For the prevention model of colitis, mice were given PA, once daily, at different doses (0.01 mg/kg, 0.1 mg/kg, 0.5 mg/kg and 1 mg/kg diluted in ddH_2_O) by oral gavage for two weeks (Supplementary Table 1). The lowest dose was calculated based on our previous experiments with AG and HAG. Starting on day 7, mice were given 2% DSS in drinking water to induce colitis. For the colitis treatment experiments, mice were given 2% DSS in their water for 2 weeks. Starting on day 7, mice were given PA at the same doses as the prevention experiments (0.01 mg/kg, 0.1 mg/kg, 0.5 mg/kg and 1 mg/kg) by oral gavage. Control mice were given ddH_2_O by oral gavage (Supplementary Table 1). The weight of mice was monitored over the duration of the experiment. The mice were sacrificed on day 14, colons were harvested, their length was measured, and they were processed for further analysis.

Blood in stool was detected using Hemoccult (Beckman Coulter) fecal immunochemical test. Immediately before sacrifice, stool consistency (0-fully formed stool; 2-loose stool; 4-diarrhea) and blood in the stool (0-no blood; 2-detected using Hemoccult; 4-rectal bleeding) were scored, and these measurements were used along with the weight difference in mice from the beginning to the end of the experiment (0 = no weight loss; 1 = 0–5% weight loss; 2 = 6–10% weight loss; 3 = 11–15% weight loss; 4 = 16–20% weight loss), to calculate the CDI.

### Immunohistochemistry

Sections of paraffin-embedded colons were incubated with cyclooxygenase-2 (COX-2) (cat #60126; Cayman Chemical Company, Ann Arbor, MI) mouse polyclonal antibody, diluted 1:10,000 in Antibody Amplifier™ (ProHisto, LLC, Columbia, SC) overnight. The slides were then processed using EnVision+ System HRP kits (DAKO, Carpinteria, CA) according to the instructions provided by the kit, which uses the chromagen, diaminobenzidine to elicit dark brown reaction to the HRP-tagged secondary antibody provided in the kit. Methyl green was used as a secondary stain. Immunoreactivity score was obtained by multiplying scores from two criteria – 1) percentage of tissue stained (0–5: 0 (0% positive staining), 1 (< 10%), 2 (11–25%), 3 (26–50%), 4 (51–80%), or 5 (> 80%)), and 2) staining intensity (0–3: 0 (Negative staining), 1 (Weak), 2 (Moderate), or 3 (Strong)). The scores of two parameters are multiplied, giving a range of scores between 0–15.

### Inflammation scoring

Colons were fixed in formalin for 24 hours, then sent to the Instrumentation Resource Facility in the University of South Carolina, School of Medicine in Columbia, South Carolina, U. S. A. Paraffin-embedded colons were serially sectioned (5 μm) and one section from each mouse was stained with hematoxylin and eosin. The stained slides were blindly examined under a microscope by two investigators for histopathological changes and scored according to a system previously described and extensively used by our lab and many others [19, 42, 43]. Briefly, the histology score for inflammation accounts for four parameters – 1) inflammation severity (0 (no inflammation), 1 (minimal), 2 (moderate), and 3 (severe)); 2) inflammation extent (0 (no inflammation), 1 (mucosa only), 2 (mucosa and submucosa), and 3 (transmural)); 3) crypt damage (0 (no crypt damage), 1 (one-third of crypt damaged), 2 (two-thirds damaged), 3 (crypts lost and surface epithelium intact), and 4 (crypts lost and surface epithelium lost)) and; 4) percentage area of involvement (0 (0% involvement), 1 (1–25%), 2 (26–50%), 3 (51–75%), and 4 (76–100%)). The scores for the first three parameters are added and the sum is multiplied by the fourth parameter, giving a range of scores between 0–40.

### Statistical analysis

Data are expressed as a mean ± standard error of the mean. Mean differences were compared by one-way analysis of variance (ANOVA), followed by Dunnett’s multiple comparison tests. A *P*-value of ≤ 0.05 was chosen for significance.

## SUPPLEMENTARY MATERIALS



## Abbreviations

AG: American Ginseng
AOM: Azoxymethane
CAM: Complementary and Alternative Medicine
CD: Crohn’s Disease
CDI: Clinical Disease Index
DSS: Dextran Sulfate Sodium
IBD: Inflammatory Bowel Disease
HAG: Hexane fraction of American Ginseng
IFN: Interferon
mФ: Macrophages
PA: Panaxynol
UC: Ulcerative Colitis
