# Supplementary material for: Panaxynol, a bioactive component of American ginseng, targets macrophages and suppresses colitis in mice
Source: Oncotarget. 2020 Jun 2;11(22):2026–36. doi: 10.18632/oncotarget.27592 (PMC7275787; doi:10.18632/oncotarget.27592)
Supplement: Supplementary file 1 [file oncotarget-11-2026-s001.pdf]

Panaxynol, a bioactive component of American ginseng, targets macrophages and suppresses colitis in mice

SUPPLEMENTARY MATERIALS

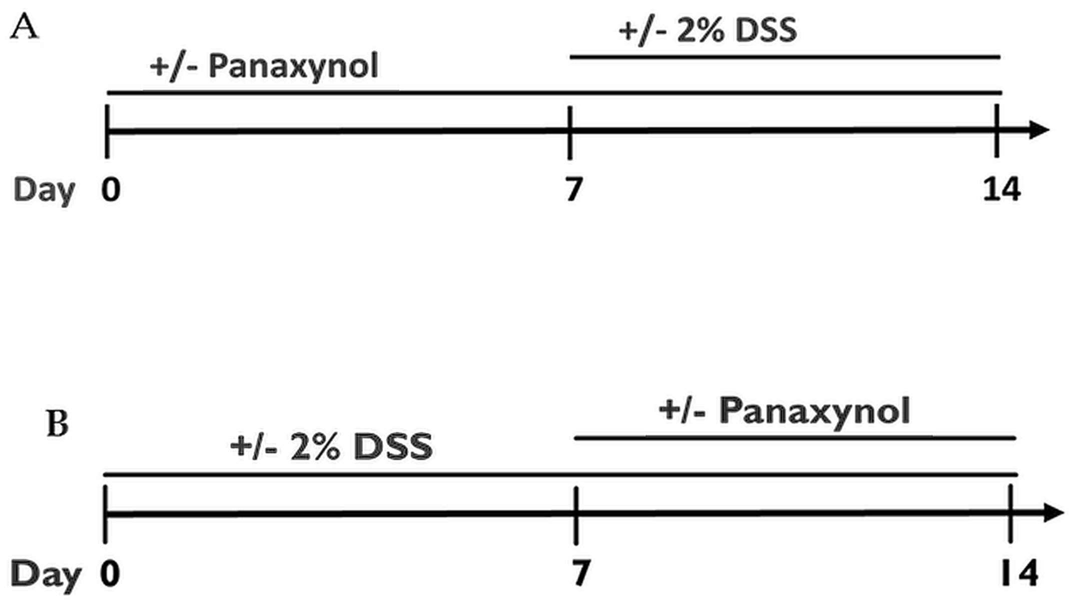

Supplementary Figure 1: Schematics of *in vivo* experimental courses. (**A**) Prevention model of colitis where the mice were treated with Panaxynol before induction of colitis using 2% DSS. (**B**) Treatment model where mice were treated with Panaxynol after the manifestation of the disease due to DSS treatment.

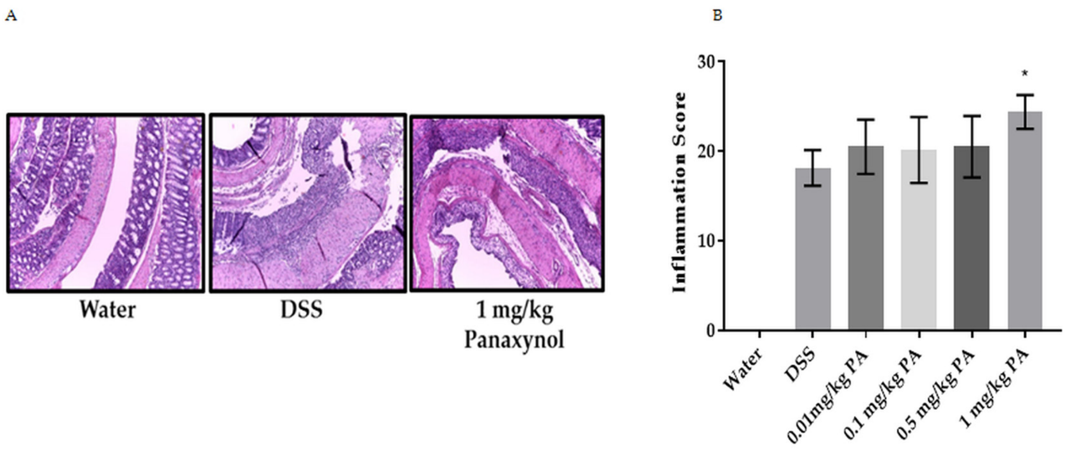

Supplementary Figure 2: Panaxynol does not prevent colitis in mice. (**A**) Representative images of H & E stained sections of colons (100×). (**B**) Inflammation scores obtained from H & E slides of the colon cross-sections.

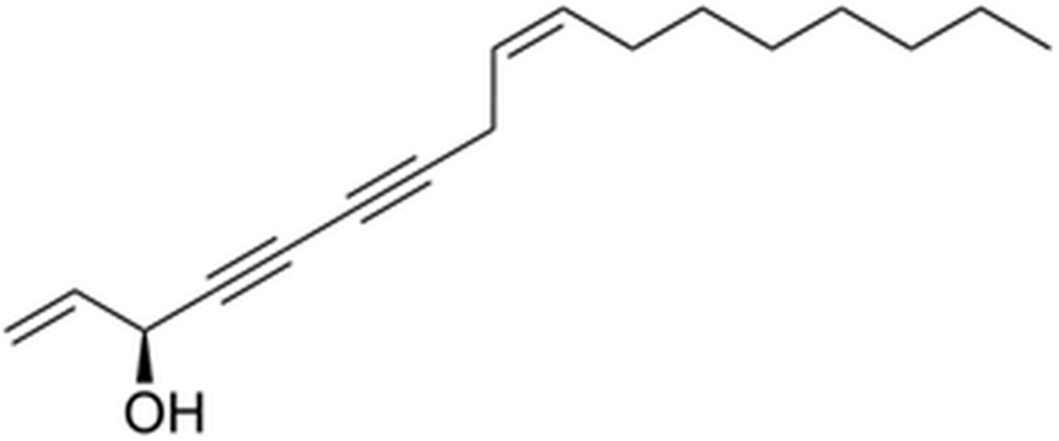

Supplementary Figure 3: Structure of Panaxynol.

Supplementary Table 1: Treatments and conditions for each group

Group	DSS	Panaxynol
1	–	-
2	+	-
3	+	0.01 mg/kg
4	+	0.1 mg/kg
5	+	0.5 mg/kg
6	+	1.0 mg/kg

$n = 8$.
